# Production of Single-Cell Oil from a Local Isolate *Bacillus subtilis* Using Palm Fronds

**DOI:** 10.1155/2023/8882842

**Published:** 2023-11-01

**Authors:** Wassan Dawood Musa Al-Obeidi, Dhafer F. Al-Rawi, Loay H. Ali

**Affiliations:** College of Education for Pure Sciences, University of Anbar, Ramadi, Iraq

## Abstract

This study, conducted at the Department of Biology, University of Anbar, Iraq, focuses on addressing the escalating issue of contamination and aims to acquire microbial oils to alleviate the global shortage in plant and animal oil production, utilizing environmental waste as a carbon source to reduce global pollution and select efficient local bacterial isolates of *Bacillus subtilis* for the production of single-cell oil (SCO) using local soil and environmental waste as a carbon source. Four isolates were selected as the best in producing single-cell oil, with the isolate with code C4 standing out as it recorded the highest production. It is worth noting that all these isolates belong to the bacteria type *Bacillus subtilis*. Palm fronds were found to be the most suitable environmental residue for SCO production compared to other waste materials (wheat straw and wheat bran). Submerged cultures were used to improve SCO production, with optimal conditions determined as pH 7, a temperature of 30°C, carbon source concentration of 3 g/100 ml, inoculum volume of 3 ml/100 ml, inoculum density of 20 × 10^7^ cells, and an incubation period of 72 hours. The Soxhlet extraction method was used to obtain the oil, which was found to contain high percentages of unsaturated fatty acids, particularly linoleic acid (46.030%) and palmitoleic acid (16.579%). The oil was highly soluble in chloroform and ethanol but insoluble in water. The saponification test indicated the potential for soap production from the oil. This comprehensive research addresses the need for locally sourced and sustainable SCO production, offering insights into the selection of efficient bacterial isolates, the optimization of cultivation conditions, and the valuable properties of the resulting SCO. The significance of this study lies in the production of single-cell oil from soil-isolated *Bacillus subtilis* bacteria for use in food applications.

## 1. Introduction

The industrial revolution and the rapid development of agricultural technologies led to the introduction of a multitude of pollutants into our daily lives [1], which, over time, began to create environmental problems. It was believed that nature could overcome these environmental problems by itself, but the increasing amount of waste has slowly begun to exceed the limits that can be naturally absorbed [2].

Currently, environmental research focuses on mitigating the environmental damage caused by waste build-up [3] with regard to the by-products of agricultural industries, considering them as intermediates to the biological production of high-value products, which represents a viable alternative to deal with the environmental impacts of waste [4–6].

The use of microorganisms in ecosystems can help reduce waste and create a pollution- and toxin-free environment [7].

Oily microorganisms can produce single-cell oil (SCO) through the use of different carbon sources in their growth media [8]. Industrial applications that make use of these organisms are somewhat limited in number, such as ingredients added to cosmetics and using cooking oil to produce biofuel; however, due to the high cost of production, but the combination of SCO production and other biotechnology applications, including waste and by-products, has overcome this particular difficulty [9].

Oils derived from microbial sources are called microbial oils or SCO oils [10].

SCOs are intracellular lipids composed of TAGs, free fatty acids, polar lipids, sterols, hydrocarbons, and pigments [11, 12]. The term SCO is used as a parallel to SCP to refer to oils of microbial origin [13].

All microorganisms contain lipids, usually at about 6 to 8% (w/w) of the dry cell weight [14]. Microorganisms that accumulate more than 20% of their weight as fat on a cell dry weight basis are considered oily microorganisms [15], a definition that includes various types of microalgae, bacteria, yeasts, and molds capable of accumulating fat.

Oily microorganisms can accumulate lipids and predominant tri-glycerol and fatty acids more efficiently than plants in a shorter production cycle that gave yields ranging from 20 to 87% of their dry weight [16].

Microorganism-derived oils, also known as single-cell oils (SCOs), are similar in composition to vegetable oils [17] and animal fats [11]; however, SCOs are preferred over oils derived from plants and animals because it is easy to increase their production.

Also, SCO production is unaffected by factors such as seasonal changes, geographic location, harvest time, and transportation, which are a concern when relying on plant and animal materials [18].

The propensity of microorganisms to accumulate lipids is mainly determined by their genetic characteristics [19], and this may differ between species or even between strains of a particular species [20].

Several types of bacteria were used to produce SCOs, including *Bacillus subtilis*, *Rhodococcus opacus*, *Streptomyces* sp., and *Acinetobacter baylyi* [21].

*B. subtilis* bacteria, also known as grass bacillus or hay bacillus, are mostly found in soil [22] and are not pathogenic to humans [23]. *B. subtilis* bacteria can be isolated from many terrestrial and aquatic environments, which make this species apparently omnipresent; however, this type of bacteria is the same as all other bacilli that can form inactive spores with high durability that enables them to resist nutritional deficiency and unfavorable environmental conditions [24].

The study aims to produce single-cell oils SCO from local bacterial *B. subtilis* isolates using available plant waste in the Iraqi local environment. Additionally, it aims to identify the fatty acid content of these plant oils.

## 2. Methodology

### 2.1. *B. subtilis* Isolate

Twenty samples were collected from the soil of the rhizosphere of different plants that were selected from separate areas in Anbar Governorate for the purpose of isolating *B. subtilis* bacteria.

A series of decimal dilutions of the selected soil samples was prepared by adding 10 grams of each soil sample after cleaning out impurities to 90 milliliters of sterile water in a glass flask with a capacity of 250 milliliters and then placed in a water bath at 80°C for 20–30 minutes to degrade any vegetative cells accompanying the *B. subtilis* spores, after which a series of decimal incisions were made.

The method of pouring plates was used, as 1 ml of the fourth dilutions, 10^−4^, and the fifth, 10^−5^, were taken, each of which was placed in a sterilized Petri dish along with the isolation medium, consisting of 10 g tryptone, 5 g yeast extract powder, 10 g NaCl, and 15 g Agar in 1000 ml of distilled water. The plates were incubated at 30°C for 24 hours, after which the growing isolates were purified by subculturing them on the same used culture media. The process was then repeated several times to obtain pure cultures and single colonies. To ensure that the developing isolates belonged to the genus *B. subtilis*, they were plotted on the selective medium (HiCrome^TM^ Bacillus Agar Base) prepared according to the manufacturer's instructions. The dishes were incubated at 30°C for 24 hours, after which the appearance of bacterial colonies in a light green color on this medium indicates the presence of *B. subtilis*.

### 2.2. Identification of *B. subtilis* Isolates

The obtained bacterial colonies were identified based on their morphological characteristics including colony shape, texture, height, color, and edges. The characteristics of the bacterial cells were then studied microscopically after staining them with Gram's stain according to Holt et al. [25]. To ensure the formation of spores, they were stained with Malachite green. Biochemical tests were also conducted for the bacterial isolates in order to diagnose them. These tests included the following:

Oxidase test, as per Baron et al. [26]; catalase test, as per Baron et al. [26]; motility test, as per Murray et al. [27] and Forbes et al. [28]; Voges–Proskauer test, as per Colle et al. [29] and MacFaddin [30]; methyl red test, as per Colle et al. [29] and MacFaddin [30]; indole test, as per Cruickshank et al. [31]; urease test, as per MacFaddin [30] and Forbes et al. [32]; citrate utilization test, as per Atlas et al. [33] and MacFadden [30]; and starch analysis, as per Benson [34].

Testing the growth of isolates in a saline medium concentration of 6.5% NaCl: the growth efficiency of bacterial isolates was tested in a saline medium with a concentration of 6.5% by replacing the percentage concentration in the medium of a previously prepared *B. subtilis* isolate with a concentration of 6.5%.

### 2.3. Testing the Ability of the Diagnosed Bacterial Isolates to Produce Oil (Primary Screening)

#### 2.3.1. The First Stage

The selected bacterial isolates were cultured on a laboratory-prepared oil agar medium consisting of 40 grams peptone, 5 grams sucrose, 5 milliliters sunflower oil, and 15 grams agar in 1000 milliliters of distilled water, by making a circle with a diameter of 1 cm in the center of the dish and incubating at a temperature of 30°C for 48 hours, after which the diameters of the bacterial colonies growing on the medium were measured.

#### 2.3.2. The Second Stage

The growing bacterial colonies were reseeded on the oil agar medium by making a circle with a diameter of 1 cm in the center of the plate and incubating at a temperature of 30°C for 24 hours only. After that, the most efficient isolates were selected, and the diameters of the colonies growing in the center of the plate were measured.

### 2.4. Testing the Efficiency of the Selected Isolates on Collecting Oil Using Sudan Black Dye B (Secondary Screening)

The selected oil-producing isolates were planted on a nutrient agar medium in the form of a circle with a diameter of 1 cm in the center of the plate and incubated at a temperature of 30°C for 24 hours. The surface of the plate was then immersed in a solution of Sudan black dye B, prepared by dissolving 0.3 grams of dye powder black Sudan in 100 ml of 70% ethanol and then being left for 30 minutes followed by gently washing the plate with 96% ethanol [35].

### 2.5. Diagnostics with Vitek 2 Compact System

The Vitec device, produced and developed by the French company bioMerieux located in the laboratory of Al-Ramadi Teaching Hospital, was used to diagnose the selected bacterial isolate after confirming it by means of biochemical tests.

### 2.6. Preparation of the Bacterial Inoculum

The selected *B. subtilis* inoculum was prepared by culturing it in a 250 ml glass beaker containing 100 ml of nutrient broth culture medium and incubating for 24 hours, after which a white thin tissue appeared on the top of the surface and a turbidity of the medium was observed, indicating the readiness of the vaccine [36]; the bacterial density in the inoculum was then estimated.

### 2.7. Election of the Best Environmental Waste as a Carbon Source for Oil Production

According to the amount of oil produced from the bacterial isolate in the media of the three liquid wastes, palm fronds and wheat straw wastes were prepared by washing to remove dirt and impurities. Then, they were air-dried in an electric oven at 65 degrees Celsius for 48 hours (except for the bran, which was already dry and clean). The three wastes were ground to pass through a 40-micron sieve. The liquid culture method was used to determine the amount of oil produced from the bacterial isolate in each of the three media used. The three liquid media were prepared by dissolving 0.1 grams of peptone, 0.24 grams of KH_2_PO_4_, 0.24 grams of K_2_HPO_4_, and 2 grams of palm fronds, wheat bran, or wheat straw.

After that, 1 ml of the bacterial inoculum was added to the liquid fronds medium, 1 ml of the bacterial inoculum was added to the liquid hay medium, and 1 ml of the bacterial inoculum was added to the bran liquid medium, each using conical flasks with a capacity of 250 ml. The flasks were incubated at a temperature of 30°C for 24 hours. After being filtered and dried in an electric oven at a temperature of 60°C for 24 hours in a Soxhlet device using petroleum ether as a solvent to extract the oil, the percentage of oil produced from the bacterial isolate was extracted in each medium.

### 2.8. Determination of Optimal Conditions for Single-Cell Oil Production from *B. subtilis*

The liquid medium of palm fronds (single-cell oil production medium) was used to determine the factors affecting the production of single-cell oil, as will be explained in the following sections, using conical flasks of 250 ml capacity, where 100 ml of liquid medium were placed in each.

#### 2.8.1. pH

To determine the optimal pH for single-cell oil production, the pH of the medium was set at 10, 9, 8, 7, 6, and 5.

#### 2.8.2. Temperature

To determine the optimal temperature for the production of single-cell oil in the production medium, different temperatures (45, 40, 35, 30, 25, and 20°C) were used.

#### 2.8.3. Inoculum Volume

To determine the optimal inoculum size for single-cell oil production, different inoculum volumes were used (5, 4, 3, 2, 1, and 0.5 ml/100 ml of the liquid production medium).

#### 2.8.4. Concentration of Carbon Source

Palm fronds were used as a carbon source to produce single-cell oil at different concentrations (1, 2, 3, 4, 5 g/100 ml) in the medium to determine the optimal concentration for oil production.

#### 2.8.5. Incubating Time

To study the effect of the incubation period on the production of single-cell oil, the selected isolates were grown for different time periods (24, 48, 72, and 96 hours), as the isolate was grown in 100 ml of liquid medium, each separately, at the optimum temperature, pH, carbon source concentration, and inoculum size identified in the previous steps.

The percentage of oil produced in each step was estimated using the Soxhlet device according to the method described in A.O.A.C. (1990).

### 2.9. Developing the Selected Bacterial Isolate According to the Optimal Conditions for Production

The selected oil-producing bacterial isolates were developed according to the optimum conditions obtained for single-cell oil production, as determined in the previous sections.

### 2.10. Confirmation Tests for the Oil Produced

#### 2.10.1. Solubility Test

A certain volume of water, ethanol, and chloroform was placed in three separate glass test tubes, and the oil produced was then added in an amount of 1 ml to each tube, after which the tubes were then stirred. Thereafter, the melting nature of the oil produced was observed for each tube [35].

#### 2.10.2. Saponification Test

2 ml of NaOH sodium hydroxide solution (at a concentration of 2%) was added to 3 ml of the oil produced in a test tube to observe the formation of a soapy solution [35].

### 2.11. Estimation of Fatty Acids in the Oil Produced

Long-chain fatty acids were estimated via the GC device (Perkin Elmer) according to the standard method of Raslan [37]. The flow rate was 1 ml/min and 105°C for 1 minute and then the temperature was fixed to 220°C with 4 psi, whilst short-chain fatty acids were estimated according to the standard method of Torii et al. [38]. Using 1 gram of the oil, 3 ml of hexane was added, and the mixture was shaken for three minutes. Afterward, it was centrifuged for ten minutes at a speed of 10,000 revolutions per minute. The supernatant layer was withdrawn and dried with nitrogen gas. Then, 1 ml of methanol was added, and it was injected into an HPLC system at a flow rate of 0.8 ml per minute and a temperature of 30°C.

## 3. Results and Discussion

### 3.1. *B. subtilis* Isolate

In our study, *B. subtilis* was isolated from the soil of the rhizosphere of different plants, as the bacteria belonging to the genus *Bacillus* are classified as rhizobacteria that promote plant growth and are found in the rhizosphere [39].

**Figure 1:**
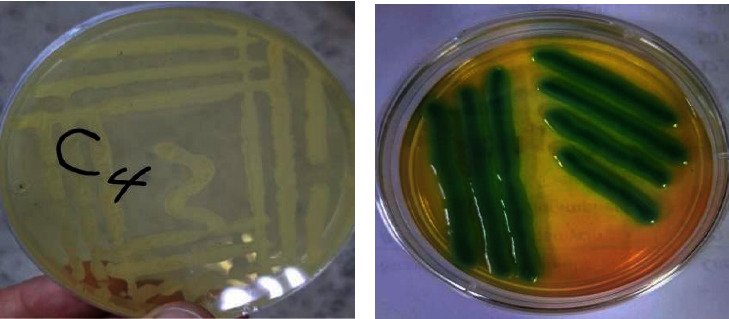
Bacteria growth. (a) Growth of bacterial isolates on the selective medium prepared in the laboratory. (b) Growth of bacterial isolates on the prepared selective medium.

Al-Rawi and Qadir [40] and Al-Attar et al. [41] indicated the possibility of isolating *B. subtilis* from soil. 15 bacterial isolates were obtained that grew with high efficiency on the isolation medium (Figure 1(a) out of 20 soil samples brought to the laboratory, where the isolation percentage was 75%. Also, all bacterial isolates grew on the prepared selective medium (HiCrome^TM^ Bacillus Agar Base) as indicated by their light green color Figure 1(b).

### 3.2. Identification of *B. subtilis* Isolates

#### 3.2.1. Culturing and Microscopic Characteristics

The cultivation characteristics of the bacterial isolates grown on the selective medium were studied, from which it was found that most of the colonies of these bacterial isolates are circular in shape with different regular and irregular edges (some of them have zigzag or lobed edges) and are white-yellow in color and medium to large in size.

**Figure 2:**
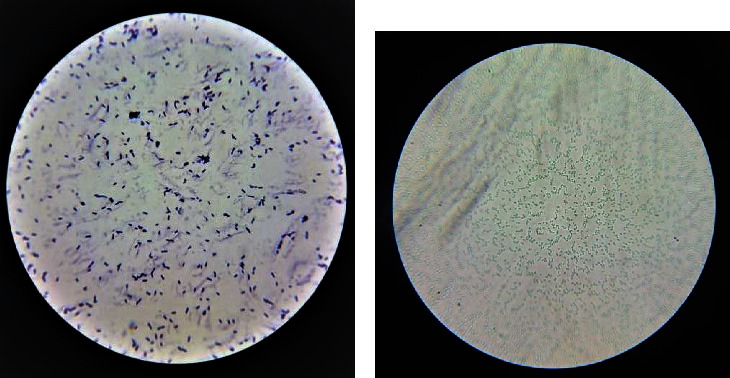
Microscope photos of *B. substiles* cells. (a) *B. subtilis* cells under a compound light microscope (100x magnification), after staining them with Gram stain. (b) Formation of blackboards after 30 days, from keeping *B. subtilis* in the refrigerator.

The cells' shapes and arrangement after being stained with Gram's stain showed that they are sticky, straight, or oblong and positive for Gram's stain, often in the form of pairs or chains Figure 2(a), while staining them with Malachite green showed that they are spore-forming Figure 2(b). From these characteristics, they were found to match the characteristics of the *B. subtilis* bacterium [42].

#### 3.2.2. Biochemical Tests

The results of the biochemical tests adopted in the diagnosis of bacterial isolates are shown in Table 1.

### 3.3. Testing the Ability of the Diagnosed Bacterial Isolates to Produce Oil

#### 3.3.1. Primary Screening (First Stage)

The results of the initial screening reported in Table 2 indicate the growth of all bacterial isolates (15) from different places with the same condition on a medium agar of oil that was used as a source of carbon and energy. The same table shows a discrepancy in the diameters of the growing colonies. This could be due to the variation in the ability of these isolates to consume oil as their only source of carbon and energy, as well as the nature of the source of isolation for bacteria.

The same table also shows that the largest diameter of the growing colonies was 8.5 cm, whilst the lowest was 1.5 cm.

Through these results, the eight most efficient bacterial isolates in growth were selected for the purpose of screening them during the second stage, which are K3–L3–K3/1–C 4–E 4–M 4–F4/1–D 4/1.

#### 3.3.2. Primary Screening (Second Stage)

Table 3 shows the results of the second stage of the initial screening. The results showed that four highly efficient isolates were obtained. The diameter of the colonies for these isolates was 8.5 cm after 24 hours of incubation on average. These isolates are E 4–C 4–D4/1–F 4/1.

### 3.4. Testing the Efficiency of the Selected Isolates to Collect Oil Using Sudan Black Dye B (Secondary Screening)

**Figure 3:**
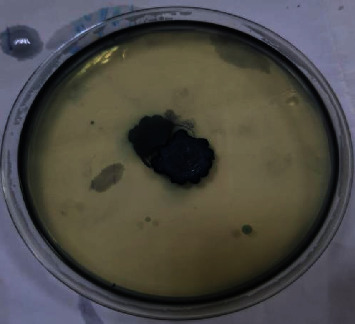
Isolate C4 stained with Sudan black dye.

The results showed that the isolate with the symbol C4 was the most efficient in its ability to accumulate oil (Figure 3).

Osman et al. [43] used Sudan black dye B to examine the ability of *Rhodotorula diobovata* yeast strains to produce oil.

### 3.5. Diagnostics Vitek 2 Compact System

The results of the diagnostic tests on the Vitec device confirmed that the isolate with the local code C4 belongs to the type *B. subtilis*, with a probability of 91%.

### 3.6. Choosing the Optimal Environmental Waste as a Carbon Source for Oil Production

**Figure 4:**
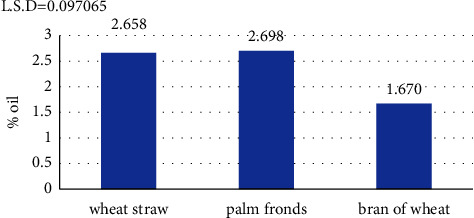
Percentage oil production according to type of plant waste used.

The results in Figure 4 regarding the proportions of oil obtained using the three previously mentioned agricultural wastes and by the method of liquid farms showed that there is a discrepancy in oil production, as the highest production was 2.698% using palm fronds, while the lowest was 1.670% using wheat bran.

The discrepancy in the oil production according to the source used may be due to the fact that palm fronds contain a high proportion of fat in the form of lignin, which helps to increase the percentage oil collection by the selected bacterial isolate; the high ratio of C : N and the low proportion of nitrogen in palm fronds are important reasons for the increased proportion of oil so produced. The C : N ratio is considered one of the most important factors in the production and collection of microbial fats [16, 44].

It should be noted that to the best of our knowledge, there is no previous study in this field with which direct comparison can be made.

### 3.7. Effect of Optimum Conditions on SCO Production

#### 3.7.1. pH

**Figure 5:**
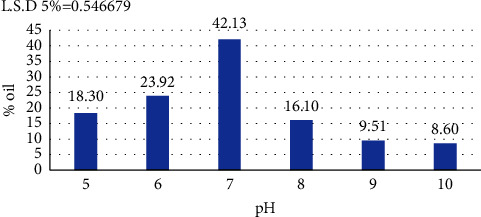
The effect of pH on the oil produced from *B. subtilis* isolate.

The results shown in Figure 5 indicate that the highest proportion of oil was produced at pH 7, which amounted to 42.13% of the biomass, followed by pH 6 at 23.92%; oil productivity decreased significantly at other pH values. These results are consistent with those of Siammai et al. [45] who also noted that the best SCO production from *B. subtilis* was at pH 7.0 and with those of Rasouli et al. [21] who also reported that pH 7.0 was the optimum in production for the bacteria *Kocuria* sp.; by contrast, Khaleel and Al-Rawi [46] indicated that pH 10 was the optimal pH for the production of oil from the bacterium *Pseudomonas fluorescens*.

In this regard, Mironov et al. [47] indicated that pH has a clear effect on oil production rates as it is a stress factor for cell growth and affects the aggregation of oils and the formation of fatty acids. pH also affects various metabolic processes, including nutrient solubility and absorption, and the activity of enzymes responsible for oil production.

#### 3.7.2. Temperature

**Figure 6:**
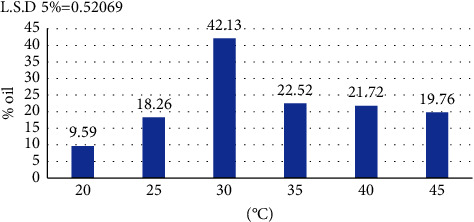
The effect of temperature (°C) on oil production by *B. subtilis* isolate at pH 7 inoculum volume.

Figure 6 shows that the highest oil production for the selected *B. subtilis* bacteria was at a temperature of 30°C, for which the proportion of oil produced was found to be 42.13% of the biomass, followed by a temperature of 35°C, where oil production was 22.52%; production was found to decrease significantly at other temperatures. These results are consistent with those of Rasouli et al. [21], who reported that the optimal temperature for single-cell oil production from *Kocuria* sp. was 30°C, and Khaleel and Al-Rawi [46] who indicated that the highest SCO production from *Pseudomonas fluorescens* was, again, at a temperature of 30°C.

Temperature is an essential and influencing factor in the growth and vital activities of microorganisms, as well as an essential means for controlling metabolic activities, both structural and destructive, especially in fermentation processes [48].

A majority of studies indicate that there is a clear effect of temperature on the production of unicellular oils and the synthesis of unsaturated fatty acids through its effect on the cellular membranes and their fluidity, since the latter affects the work of membrane-bound enzymes and transport mechanisms to and from the cell [47].

#### 3.7.3. Inoculum Volume

**Figure 7:**
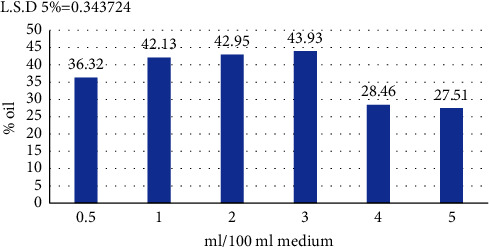
Effect of amount of inoculum (ml) on oil produced by *B. subtilis* isolate at pH 7 and a temperature of 30°C.

The results in Figure 7 show that the highest oil productivity was when the bacterial inoculum was added at a volume of 3 ml/100 ml to a production medium with a density of 20 × 10^7^ cell/ml, for which production was 43.93%, after which the production rate decreased when the inoculum volume was increased or decreased. The results we obtained agree with those of Khaleel and Al-Rawi [46] who showed that an inoculum volume of 3 ml/100 ml medium was optimal for the production of SCO from *Pseudomonas fluorescens*.

The concentration of inoculum is one of the more important factors in production, as the production process is controlled in its conditions, including the size of the inoculum used to reach the highest production [49].

The size and type of the production process determine the amount of inoculum used. This amount must be in a quantity commensurate with the amount of culture medium prepared for production. The aim is to reduce the fermentation period at the start of production [50]. Low pollen size leads to a longer imprinting phase, which in turn delays the onset of lipid accumulation and thus leads to a decrease in production [51]. Also, a low pollen size leads to a decrease in biomass production, which reduces lipid productivity [52]. As for increasing the amount of inoculum, this leads to rapid growth and depletion of nutrients, which affects the fat yield. Also, an increase in the amount of the inoculum leads to an increase in competition for nutrients in the culture medium, which causes a decrease in growth [53].

#### 3.7.4. Carbon Source Concentration

**Figure 8:**
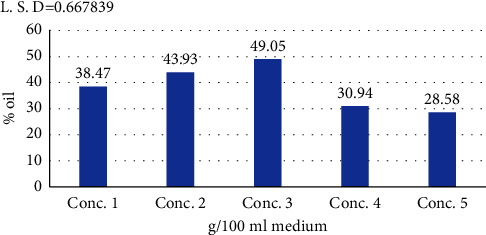
The effect of the concentration of the carbon source (palm fronds) in g/100 ml medium on the oil produced from the bacterial isolate *B. subtilis* at pH 7, a temperature of 30°C, and an inoculum volume of 3 ml/100 ml medium.

Figure 8 shows that the highest oil yield was at a concentration of 3 g/100 ml medium, as the proportion of oil produced was 49.05% after an incubation period of 48 hours and at a temperature of 30°C and a pH of 7. This decreased at other concentrations to its lowest of 28.58% at a concentration of 5 g palm fronds/100 ml medium. The important factor on which single-cell lipid formation depends is on the availability of nutrients that lead to a large amount of carbon source to stunted growth and oil production even if nitrogen is present in less quantity.

There is a need for further knowledge and experience in selecting an appropriate carbon source and determining an appropriate concentration. The relationship between the rate at which the carbon source is added and fat accumulation is positively linked. However, high concentrations of carbon source actually inhibit production of oil from microorganisms.

The C : N ratio is also of great importance in the production of microbial oils [44].

### 3.8. Incubation Period

**Figure 9:**
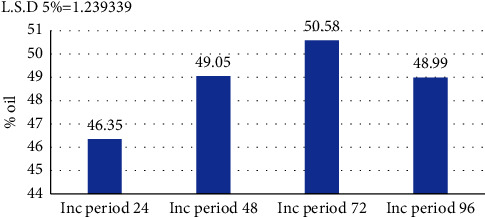
The effect of the incubation period (hours) on the oil produced (%) from an isolate of *B. subtilis* at pH 7, a temperature of 30°C, an inoculum volume of 3 ml/100 ml medium, and a carbon source concentration of 3 g/100 ml medium.

In order to determine the best incubation period for the fermentation medium, time periods ranging from 24 hours to 96 hours were adopted. The results shown in Figure 9 show that the highest single-cell oil production by the selected bacterial isolate was after 72 hours of incubation, where the production rate reached 50.58% at a temperature of 30°C and a pH of 7, while oil production notably decreased when the incubation period was reduced to less than 72 hours.

It was also noted that production decreased after 92 hours of incubation to 48.99%. The incubation period that we obtained differs from that reported by Zhang et al. [54] who indicated that the incubation period of 48 hours was optimal in the production of SCO from *B. subtilis* bacteria after growing them on cotton stalks as the only source of carbon and from that reported by Khaleel and Al-Rawi [49] who indicated that the best unicellular oil production from *Pseudomonas fluorescens* using corn cobs as a source of carbon source was also after 48 hours of incubation.

The duration required for incubation is related to a number of factors such as the type of isolate selected, pH, temperature, amount of inoculum, and composition of the production medium [55]. By increasing the incubation period, oily organisms can accumulate additional biomass. This leads to an increase in fat productivity that is dependent on the availability of nutrients and the specific conditions of cultivation [56]. Productivity can decrease with increasing incubation period due to nutrient depletion, changes in the medium such as pH, autolysis of cells, and the release of toxins into the medium [57].

Figure 9 shows that the optimal incubation period for production is 72 hours, which is sometimes considered quite a long period. This may be due to the nature of the carbon source used (palm fronds), since this source contains a high proportion of lignin. Bacteria need a longer period and the ability to secrete specialized enzymes for the purpose of its analysis. Note that this source led to a higher productivity than SCO.

### 3.9. Developing the Selected Bacterial Isolate According to Optimal Conditions for Production

The selected bacterial isolate was grown according to all optimal conditions that were obtained from pH, temperature, volume of inoculum, carbon source concentration, and incubation period, as shown in Table 4. The proportion of oil production after setting all conditions to optimal was 51.32%.

This result differs from that of Rasouli et al. [21] who achieved a 24.57% production of unicellular oil from *Kocuria* sp. while being similar to that achieved by Khaleel and Al-Rawi [46] who obtained a 55.47% production of SCO from *Pseudomonas fluorescens*.

### 3.10. Confirmation Tests for the Oil Produced

**Figure 10:**
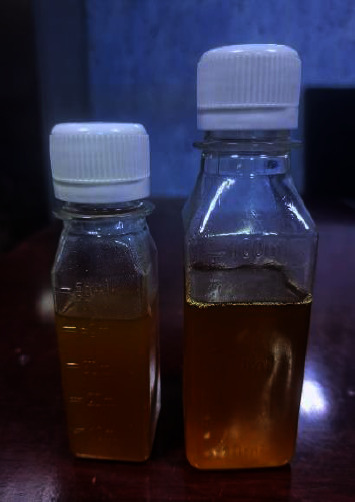
Oil produced from *B. subtilis*.

The oil produced from *B. subtilis* was liquid at room temperature, with a brownish-yellow color, as shown in Figure 10. Confirmation tests were conducted, including the following.

#### 3.10.1. Solubility Test

**Figure 11:**
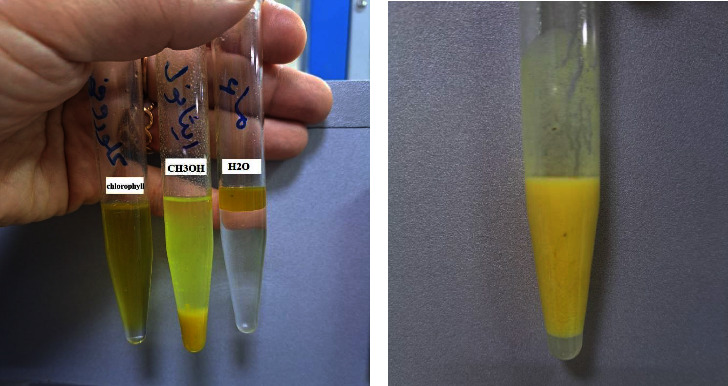
Different tests performed. (a) Solubility test. (b) Saponification test.

The results of the solubility test conducted on the oil produced shown in Figure 11(a) indicated that the oil had a high solubility in chloroform solution and a lower solubility in ethanol, while it did not show any solubility in water.

Oils have been identified as a variety of hydrophobic compounds that are soluble in nonpolar organic solvents such as ether and chloroform, which are insoluble in water [58].

#### 3.10.2. Soaping Test

The results of the saponification test conducted on the oil produced from *B. subtilis* illustrated in Figure 11(b) showed that a soapy solution was formed.

This is because any type of fat or oil can be converted into soap by reacting with an aqueous base such as sodium hydroxide or potassium hydroxide [59].

### 3.11. The Fatty Acid Content of the Oil Produced

The results of analysis of long-chain fatty acids using the GC device and short-chain fatty acids using the HPLC device reported in Table 5 A and B, respectively, indicated that the oil contained a high proportion of unsaturated linoleic acid, at 46.03%.

Cianchetta et al. [16] concluded that linoleic acid and stearic acid are among the most important fatty acid constituents of the unicellular oil produced from *Cutaneotrichosporon oleaginosum*, *Rhodosporidiobolus azoricus*, and *Lipomyces starkeyi*. Khaleel and Fakhri [60] obtained SCO with high linoleic acid and stearic acid content, while Burgstaller et al. [61] noted that the main fatty acid present in the oils produced from Apiotrichum brassicae and *Pichia kudriavzevii* was oleic acid.

## 4. Conclusion

Isolating strains of *Bacillus subtilis* from local agricultural soil efficiently produced SCO. The utilization of local environmental waste shows the potential for obtaining high yields of SCO. The produced oil composition contains a high proportion of unsaturated fatty acids. These findings highlight the promising potential of using local resources for sustainable single-cell oil production.

### 4.1. Recommendations

The recommendations are exploring new methods for extracting monounsaturated oil, increasing the extracted oil's percentage, and conducting further studies to isolate fatty acids according to their types from the produced oils.

## Figures and Tables

**Table 1:** The results of the biochemical tests adopted in the diagnosis of bacterial isolates.

Order	Isolate code	Oxidase	Catalase	Motility	Simmons citrate	Urease	MR	VP	Indole	Starch analysis	Growth in 6.5% NaCl
1	K_3_	−	+	+	−	+	+	−	−	+	+
2	L_3_	−	+	+	+	+	−	+	−	+	+
3	A_3_	−	+	+	−	−	+	−	−	−	+
4	K_3/1_	−	+	+	+	+	+	−	−	+	+
5	N_4/1_	−	+	+	+	+	+	−	−	−	+
6	N_4/2_	−	+	+	+	+	+	−	−	+	+
7	B_4_	−	+	+	+	+	+	−	−	+	+
8	M_4_	−	+	+	−	−	+	+	−	+	+
9	C_4_	−	+	+	−	−	−	+	−	+	+
10	E_4_	−	+	+	+	−	+	−	−	+	+
11	F_4/1_	−	+	+	−	−	+	+	−	+	+
12	F_4/2_	−	+	+	−	+	−	−	−	+	+
13	D_4/1_	−	+	+	−	+	+	+	−	+	+
14	D_4/2_	−	+	+	−	+	−	−	−	+	+
15	G_4/2_	−	+	+	−	+	+	+	−	−	+

− means negative for the test and + means positive for the test.

**Table 2:** Average diameters of colonies growing on oil agar media after 48 hours.

Order	Sample code	Diameter of the developing colony (cm) 48 hours later
1	K_3_	8.5
2	L_3_	8.5
3	A_3_	2.1
4	K_3/1_	8.5
5	N_4/1_	2
6	N_4/2_	1.5
7	B_4_	1.6
8	M_4_	6.6
9	C_4_	8.5
10	E_4_	8.5
11	F_4/1_	8.5
12	F_4/2_	1.5
13	D_4/1_	8.5
14	D_4/2_	1.7
15	G_4/2_	2.2

**Table 3:** The average diameter of the colonies growing on the oil agar medium after 24 hours.

Diameter of the developing colony (cm) 24 hours later	Sample code	Order
8.5	E_4_	1
8.5	D_4/1_	2
8.5	C_4_	3
8.5	F_4/1_	4
7.5	K_3_	5
6.5	L_3_	6
5	K_3/1_	7
7	M_4_	8

**Table 4:** The development of the bacterial isolate *Bacillus subtilis* according to the optimal conditions for production.

The percentage of oil production (%)	Incubation period (hr)	Inoculum size (milliliters)	Carbon source concentration (g)	Temperature (°C)	pH
51.32	72	3	3	30	7

**Table 5:** Fatty acids present in the oil and the proportions of (A) long chain and (B) short chain.

Order	A		B	
	Fatty acid	Percentage (%)	Fatty acid	Percentage (%)
1	Palmitic acid (saturated)	6.383	Propionic acid (unsaturated)	8.914
2	Palmitoleic acid (unsaturated)	16.579	Acetic acid	1.723
3	Stearic acid (saturated)	1.996	Butyric acid (saturated)	0.663
4	Oleic acid (unsaturated)	4.449	Formic acid	0.511
5	Linoleic acid (unsaturated)	46.030	Lactic acid	5.081
6	Linolenic acid (unsaturated)	7.66		

## Data Availability

The data used in this study are available from the corresponding author upon request.
